# Investigation of microstructural alterations of the human subchondral bone following microfracture penetration reveals effect of three‐dimensional device morphology

**DOI:** 10.1002/ctm2.230

**Published:** 2020-12-02

**Authors:** Thomas Onken, Liang Gao, Patrick Orth, Magali Cucchiarini, Rainer Maria Bohle, Stefan Rupf, Matthias Hannig, Henning Madry

**Affiliations:** ^1^ Center of Experimental Orthopaedics Saarland University Medical Center and Saarland University Homburg/Saar Germany; ^2^ Institute of Pathology Saarland University Medical Center and Saarland University Homburg/Saar Germany; ^3^ Clinic of Operative Dentistry Periodontology and Preventive Dentistry Saarland University Medical Center and Saarland University Homburg/Saar Germany

Dear Editor,

Small articular cartilage defects are commonly treated with microfracture.^1^, ^2^, ^3^, ^4^ Forcing the tip of a microfracture awl into the debrided subchondral bone creates a canal, through which migrating mesenchymal stromal cells (MSCs) induce repair.^5^ When the device passes, peri‐hole trabeculae are compacted with contiguous microfractures. Here, we investigate the effects of three‐dimensional (3D) instrument characteristics on bone microstructure. We hypothesized that (a) 3D tip design varies significantly, (b) morphological differences translate into different effects on the human subchondral bone microstructure, and (c) spatial parameters correlate with subchondral bone changes.

To test the first hypothesis, we precisely moulded the tips of nine awls and quantified their 3D parameters (Figure 1A) by dividing micro‐computed tomography (CT) images of each tip into three geometric structures (1 mm constant height). Their base diameters (D1‐3), base areas (A1‐3), individual volumes (V1‐3), and total volumes (V1±V2±V3) were determined (Figure 1B). A Kirschner (K) wire (CL Medical, Lyon, France) whose dimensions were calculated served for comparison. Nearly all tips (n = 8) were cone‐shaped, two were triangular pyramid‐shaped [Aesculap (Aesculap, Tuttlingen, Germany); K wire]. At the 1 mm distance to the tip vertex, the smallest base diameters, base areas, and volumes had both Rudolf (Rudolf Medical, Fridingen, Germany) and Linvatec light (Linvatec ConMed, New York) awls (Table S1). The largest base diameter (1.71‐fold), base area (2.97‐fold), and volume (2.9‐fold) had the Smith & Nephew large awl (Smith & Nephew, London, UK). At the distance of 3 mm, the smallest base diameter and area had the Linvatec light awl, while the smallest volume had the RZ Medizintechnik awl (RZ Medizintechnik, Tuttlingen, Germany). The largest base diameter (1.62‐fold), area (2.61‐fold), and volume (3.27‐fold) were of the Linvatec heavy awl (Linvatec ConMed, New York). Next, total tip volumes were calculated as a key measure related to the trabeculae displacement. The largest total volume was 2.61‐fold higher (Linvatec heavy awl; significantly larger than all instruments) than the smallest (Linvatec light awl; Table S2).

**FIGURE 1:**
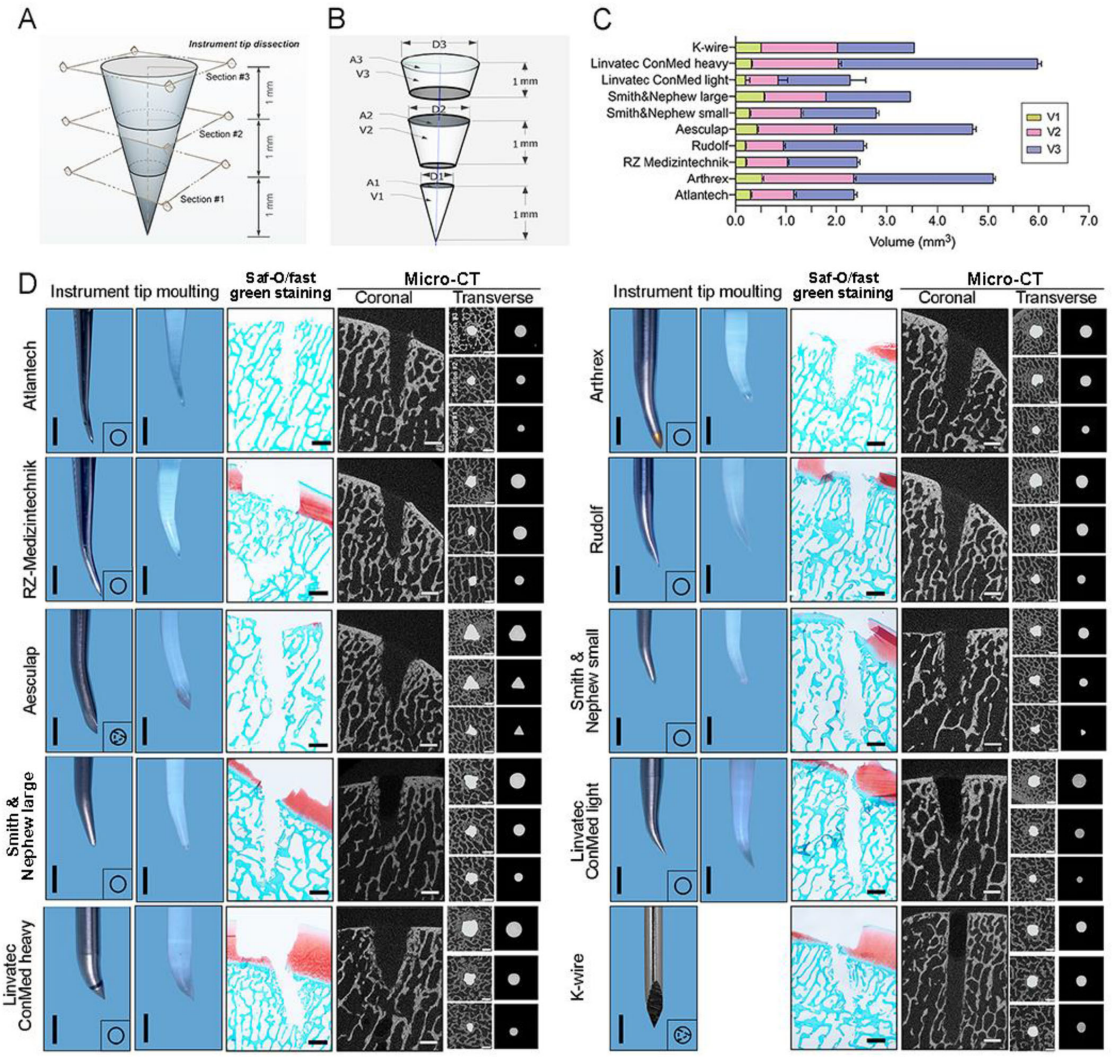
Geometric dissection, measurements, and plastic casting of tips of 10 microfracture instruments and histological and microfocused X‐ray computed tomography (Micro CT) analyses of post‐penetration microstructure of subchondral bone of cadaver knees. A, Each instrument tip was separated at sections 1‐3 into three components. B, Base diameter (D1‐3), base area (A1‐3), and individual volume (V1‐3) of each instrument tip were measured or calculated. C, Stacked bar chart showing the different volumes (V1‐3) of each instrument tip. D, Instrument tip cast and histological (Safranin O/fast green) and micro‐CT analyses (with coronal and transverse views) of post‐penetration subchondral bone. The transverse views were taken from sections 1‐3 for each instrument. Scale bar: 1 mm (instrument tip moulting); 1.5 mm (Saf‐O/fast green staining; Micro‐CT)

Simulating a clinical scenario, we next performed microfractures within rectangular full‐thickness defects (n = 10) on human medial femoral condyles (cadaveric knees) (Figure 1C; Figure S1). Qualitative appreciation of peri‐hole compaction and trabecular sealing indicated that tip morphology affected both hole geometry and peri‐canal bone microstructure [histology, quantitative microfocused X‐ray CT (micro‐CT)], irrespective of tip shapes (cone‐ or pyramid‐shaped) (Figure 1D). Bone microstructure was then quantified in two defined peri‐hole subchondral bone volumes of interests (VOIs), termed VOI1 and VOI2 (Figure 2A). To explore possible associations of morphological instrument parameters with their penetration performance, correlation analyses within both VOIs were next performed. Volumes of the instrument tips (V1, V2, V3; total volume) were positively correlated with both volume (BV/TV) and surface density (BS/TV) of bone surrounding the canals in VOI1 and VOI2 (0.139 ≤ Pearson correlation ≤ 0.694) (Figure 2B, left). These findings importantly indicate that both peri‐hole bony compaction and fracture simultaneously increases with an enlargement of the instrument tip volume. The highest correlation coefficient value for BV/TV was detected between V2 and BV/TV1 (r = 0.546, *P* = .892) (Figure 2B, middle), suggesting that especially a volume modification of the middle third critically affects peri‐hole compaction and possibly long‐term outcomes. Also, the highest correlation coefficient value for BS/TV was for BS/TV1 and total tip volume (r = 0.694, *P* = .026), indicating the tip size significantly influences the trabecular micro‐fractures induced by the instrument penetration. (Figure 2B, right).

**FIGURE 2:**
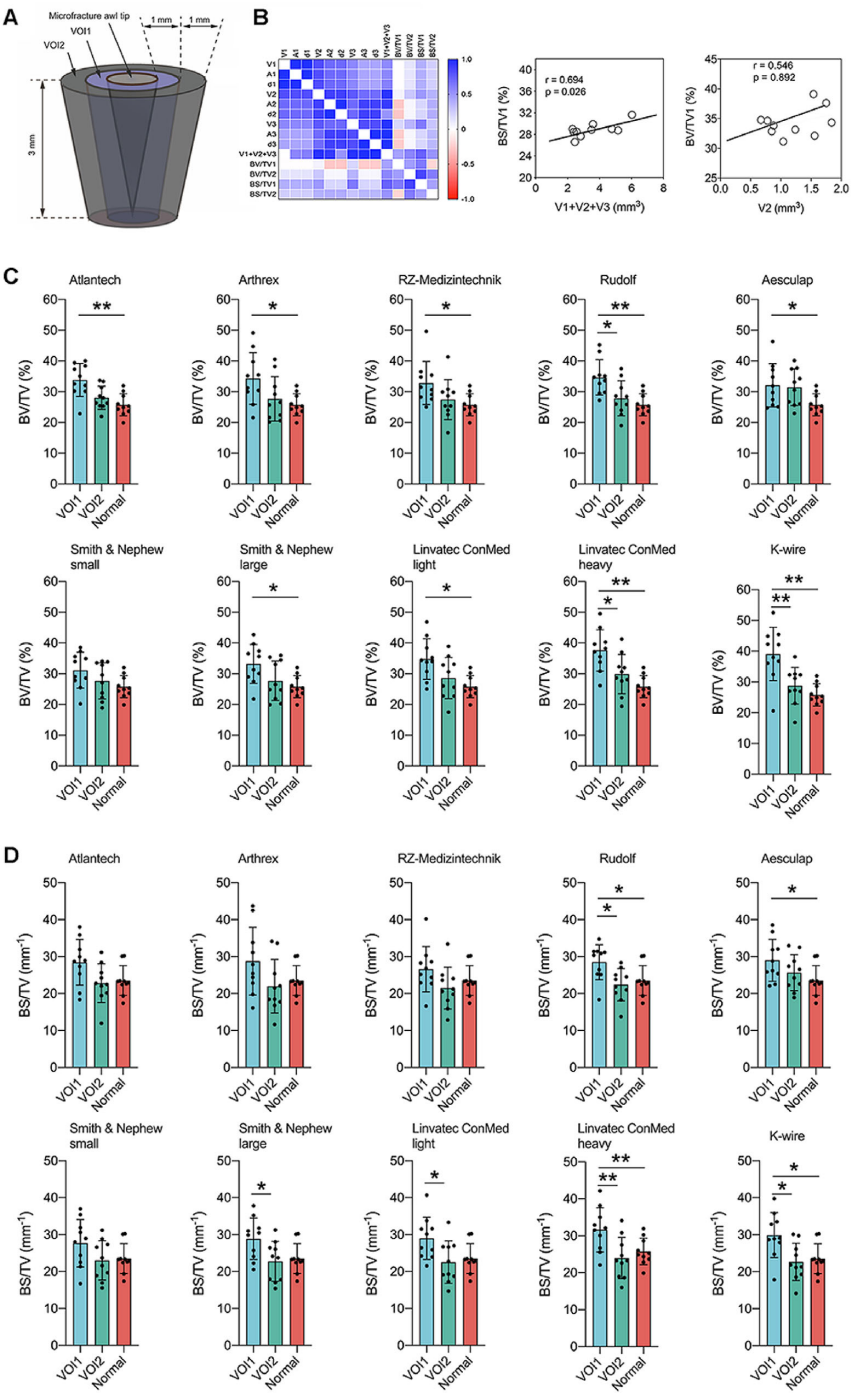
Correlation analysis between instrument parameters and subchondral bone status and quantification of subchondral bone compaction/fracture around the penetration hole. A, Volumes of interest (VOI1; VOI2) within the subchondral bone were defined as two coaxial structures surrounding the 3‐mm tip of awls. VOI1 is closer to the canal than VOI2. B, Correlation matrix of dimensional parameters of awl tip with bone volume fraction (BV/TV) or bone surface density (BS/TV) of VOI1 and VOI2. BV/TV1 and BS/TV1 were highly correlated with V2 (significantly) and V1+V2+V3 (nonsignificantly). C, Comparison of BV/TV of subchondral bone among VOI1, VOI2, and normal control. D, Comparison of BS/TV of subchondral bone among VOI1, VOI2, and normal control

We next aimed to identify the degree of microstructural bone affection following instrument penetrations by monitoring and comparing for each instrument alterations within VOI1 and VOI2. A penetration‐induced bone compaction was denoted by the significantly increased BV/TV1 when using Atlantech, Arthrex (Arthrex, Naples), RZ‐Medizintechnik, Aesculap, Smith & Nephew (large), and Linvatec (light) awls compared with normal controls (Table S2). Comparison of bone volume fraction of VOI1 (BV/TV1) or VOI2 (BV/TV2) revealed no statistically significant difference among all instruments. Increased bony compaction was reflected in a significantly increased BV/TV1 and BV/TV2 in Linvatec light and heavy awls compared with other awls (Figure 2C). No increase in either BV/TV1 or BV/TV2 compared with normal controls was noted only for the Smith & Nephew small awl (Figure 2D). Significantly higher BS/TV in both VOI1 (BS/TV1) and VOI2 (BS/TV2) than adjacent normal controls was generated by the Rudolf and Linvatec heavy awls, and K‐wire. A significantly higher BS/TV1 was caused by 1 awl (Aesculap). Many instruments did not change BS/TV in either VOI1 or VOI2 (Atlantech [Atlantech, Radevormwald, Germany]; Arthrex; RZ‐Medizintechnik; Smith & Nephew small and large; Linvatec light). Altogether, these findings advocate that mainly two effects, bone compaction and fracture, affect the subchondral bone status after microfracture. Importantly, the 3D instrument morphology determines their balance (Figure S2). A thin device leads to less bone fracture and more trabecular compaction in the adjacent VOI1. A medium‐sized instrument achieves a comparatively balanced ratio of trabecular compaction and fracture/bone loss in the adjacent VOI1. A larger tool shifts the balance of trabecular compaction and fracture towards fracture in VOI1. Our data therefore supports and extends the concept of peri‐hole subchondral bone compaction as reported in qualitative lapine studies at 1 day postoperatively, although remodeling may occur over time, similarly to a subchondral bone graft.^6^, ^7^ Future microfracture awl design should consequently consider trabecular micro‐fractures, not only their compaction. It therefore appears reasonable to use awls in clinical practice that achieve a balance between subchondral bone compaction and loss (e.g., Smith & Nephew small awl). If such an objective is to be reached, awls of smaller volume may be beneficial, as recently advocated.^8^ However, only one study so far studied, at the time of total knee arthroplasty, the capacities of a cannulated hollow awl or a conventional awl to mobilize MSCs.^9^ To the best of our knowledge, clinical effects of different awl morphologies on cartilage repair remain to be determined in patients.

Limitations include the ex vivo investigations, which do not fully simulate in vivo conditions,^10^ and selection of instruments. Strengths are the structured and detailed determination of relevant instrument characteristics, a clinically relevant setting, and robust microstructural analyses.

In sum, the most important finding of the present study is the direct influence on the human peri‐hole subchondral bone compaction as a function of device morphology. The quantitative 3D tip characteristics of the cone‐ or pyramid‐shaped instruments varied considerably. Second, these structural differences affect microfracture hole geometry and translate into different effects on the human subchondral bone microstructure, as revealed by the distinct shifts in the balance between trabecular compaction and fracture (Figure S3). Third, the total volume and the specific volume of the middle third of the awl tip positively correlates with the extent of subchondral bone alteration. Our translational findings are of clinical relevance as they may accelerate the identification of an optimal device morphology for the clinical application of treating cartilage defects in patients with microfracture. We envision further clinical studies to follow that will investigate outcome‐oriented designs of microfracture awls and joint‐specific effects of different 3D instrument morphologies on structural and clinical parameters of osteochondral repair in the long‐term in vivo.

## CONFLICT OF INTEREST

No commercial support was obtained from any company mentioned in this study. The authors disclose no financial interests and any other interests, including financial holdings, professional affiliations, advisory positions, board memberships, receipt of consulting fees, etc., which could affect either the authors’ objectivity or the content of the article.

## AUTHOR CONTRIBUTIONS

Henning Madry conceptualized and supervised the study, analyzed data, and wrote the paper. Thomas Onken, Stefan Rupf, and Matthias Hannig acquired and analyzed data. Liang Gao, Patrick Orth, Magali Cucchiarini, and Rainer Maria Bohle acquired and analyzed data, performed statistical analysis, and wrote the paper. All authors read and approved the final manuscript.

## Supporting information

Supporting information

## Data Availability

All the data obtained and/or analyzed associated with the current study were available from the corresponding authors upon reasonable request.
