# Supplementary material for: Investigation of microstructural alterations of the human subchondral bone following microfracture penetration reveals effect of three‐dimensional device morphology
Source: Clin Transl Med. 2020 Dec 2;10(8):e230. doi: 10.1002/ctm2.230 (PMC7711083; doi:10.1002/ctm2.230)
Supplement: Supplementary file 1 — Supporting information [file CTM2-10-e230-s001.docx]

**SUPPLEMENTARY MATERAILS AND METHODS**

**Instrument tips moulting**

Nine commercially available microfracture awls were obtained from Atlantech (Radevormwald, Germany; #15-1022) Arthrex (Naples, FL, USA; #AR-1761), RZ Medizintechnik (Tuttlingen, Germany; #225-600-225), Rudolf Medical (Fridingen, Germany; #AT304-001); Aesculap (Tuttlingen, Germany; #FR685R); Smith & Nephew [London, UK; #72201883 (small) and #72201884 (large)]; Linvatec ConMed [New York, NY, USA; #MFX-30LT (light) and #MFX-30HV (heavy)]. For the ease of comparison, only awls with the smallest tip from each company were tested. One triangular pyramid-tipped K-wire (diameter, 1.4 mm) (CL Medical, Lyon, France; #CL50-13-1114) was also included.

Each microfracture awl tip was marked with a 0.1 mm marker pen at three standardized distances at 1, 2, and 3 mm starting from its distal tip vertex. The instrument tip was meticulously inserted perpendicular into a moulting kit of Coltene President Microsystem (Coltene AG, Altstätten, Switzerland) at each marking line and was kept in the position for 3 min.^1^ Subsequently, the instrument was removed, generating standardized holes within the moulding system. The mouldings were filled with epoxy resin (Conrad electronics, Hirschau, Germany) and hardened at room temperature for 24 h. These molded resin structures were then used for further micro-CT analyses to determine the geometry of the instrument tips.

**Geometric measurement of instrument tips**

Each 3-mm instrument tip was analyzed by dividing the 3D micro-CT images into three structures with equal heights (each height 1 mm) (**Figure 1A**). The base diameter was recorded as the maximal transverse diameter of the instrument molds with distances of 1 mm (D1), 2 mm (D2) and 3 mm (D3) to the tip vertex, respectively (**Figure 1B**). The K-wire was regarded as an idealized cylinder with a triangular pyramid-shaped tip. For instruments with a triangular pyramid-shaped tip, the instrument base areas (A1, A2, and A3) and volumes (V1, V2, and V3) were calculated for the three structures, which were viewed as (truncated) triangular pyramids. For instruments with a cone-shaped tip, the base areas and volumes of the three structures were calculated for the three structures, which were viewed as cones or frustums. The total volume of the 3-mm instrument tip was calculated as V1 + V2 + V3.

**Cadaveric testing**

Ten fresh frozen cadaveric left-side distal femurs with visually health cartilage were obtained from Department of Pathology, Saarland University. Ethics committee approval was not required. A rectangular full-thickness defect (20 mm x 42 mm) was created on the medial femoral condyle to fit a predefined template with 10 test regions (X1-10) and 4 control regions (C1-4) (**Figure S1**). Circular regions (diameter, 4 mm) were kept at least 3 mm from either each other or defect margin, leaving the control regions undisturbed by the instrument penetrations as confirmed by micro-CT. Ten penetration holes were created at the center of the test regions by perpendicular penetration of the 10 instruments downwards to a standardized depth of 5 mm within the subchondral bone. The order of the applied instruments was always changed with a clockwise rotation to exclude the topographic effect. Reflecting the detailed measurements of each tip of 3-mm length, each penetration into the subchondral bone was analyzed to a depth of 3 mm regardless of the slope of the femoral condyle surface (**Figure 2A**).^2^

**Qualitative and quantitative evaluation of subchondral bone changes**

The subchondral bone compartment around the penetration holes and between the holes (normal controls) were separately assessed as previously described using a micro-CT scanner (Skyscan 1172; Bruker, Belgium) with a maximal nominal resolution below 0.8 μm.^3^ Two volumes of interest were defined as two coaxial truncated cone sleeves surrounding the instrument tip (VOI1; VOI2), in which VOI1 and VOI2 are 1 mm and 2 mm away from the tip/hole margin in two-dimensional micro-CT transverse views, respectively and represent as two inverted coaxial truncated cone sleeves of the awl tip in three-dimensional space (**Figure 2A**). For qualitative analysis, the compaction of subchondral bone surrounding the generated holes and sealing of the trabecular cavity were noted in both the transverse and sagittal micro-CT images. For the quantitative analysis, only bone volume fraction (BV/TV) and bone surface density (BS/TV) were determined within each of the VOIs in a three-dimensional (3D) fashion.^4^

**Correlation analysis of instrument parameters and subchondral bone status**

The 3-mm instrument tip was uniformly viewed as a right circular cone, which was additionally separated into one cone and two frustums (height, 1 mm). The parameters (base diameter, base area, and volume) of these three structures were calculated accordingly for each instrument. The Pearson correlation coefficient was calculated to evaluate the correlation between those parameters of each instrument and the corresponding BV/TV or BS/TV within the respective ROIs.

**Histological analysis**

For the histological evaluation, sections (thickness, 4 μm) were obtained using a microtome cutting from the center of each paraffin-embedded specimen and stained with safranin orange/fast green (safranin O) as previously described.^5^

**Statistical analysis**

Values are expressed as mean ± standard deviation (SD). One-way ANOVA with Tukey’s post-hoc test and Mann-Whitney U test were used where appropriate. Any *P* value < 0.05 was considered statistically significant. Calculations were performed using SPSS (IBM SPSS 20; SPSS Inc., Chicago, IL, USA).

**FIGURE S1.** Illustration of instrument penetration tests within the cartilage defect at the medial femoral condyle of cadavers. AT, Atlantech; AX, Arthrex; RZ, RZ Medizintechnik; Ru, Rudolf; AE, Aesculap; SNsm, Smith & Nephew small; SNlg, Smith & Nephew large; CML, ConMed light; CMH, ConMed heavy.

**
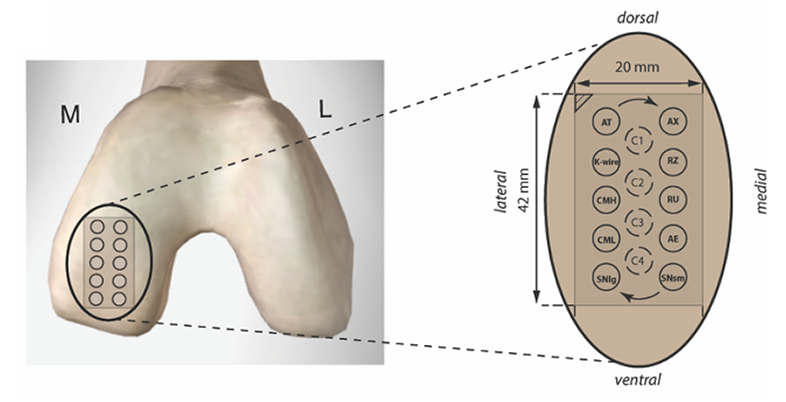
**

**FIGURE S2.** Proposed hypothetical principle of microfracture instruments affecting the balance of trabecular compaction and bone fracture. (**A**) The subchondral bone status after microfracture is determined by the balance between bone compaction and bone fracture induced by the physical penetration of instruments. The trabecular bone compaction increases bone volume fraction (BV/TV) and seals trabecular cavities, while the bone fracture increases bone surface density (BS/TV) and stimulates bone remodeling. (**B**) Illustration of the subchondral bone penetration with instruments. Of note, as the trabecular separation within the medial femoral condyle is of 0.4 -0.5 mm, all the tested instruments induce structural trabecular damage. (**C**) A thin instrument mainly leads to trabecular compaction (and resulting high BV/TV) and induces minimal trabecular fracture (BS/TV unchanged) in the adjacent peri-instrument volume of interest (VOI1); please see Table 2. (**D**) A medium-sized instrument (Smith & Nephew small awl) may achieve a balanced ratio of trabecular compaction and fracture/bone loss (both BV/TV and BS/TV are similar to normal) in the adjacent peri-instrument volume of interest (VOI1). (**E**) A large instrument shifts the balance of trabecular compaction and fracture towards bone fracture (both BV/TV and BS/TV are higher than normal) in the adjacent peri-instrument volume of interest (VOI1). BC, bone compaction; BF, bone fracture.


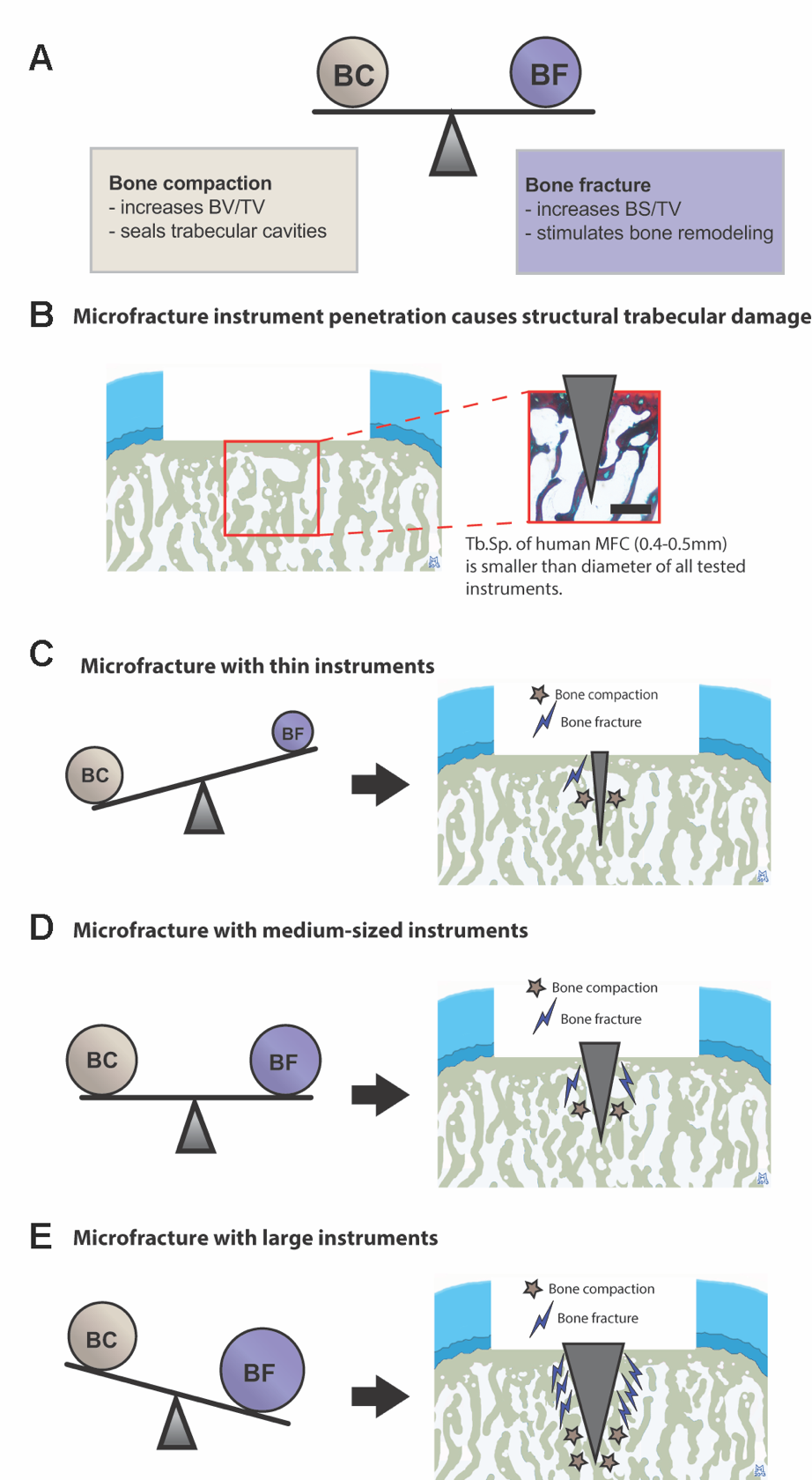


**FIGURE S3.** Correlation analysis of instrument parameters and subchondral bone status. (A) Structure of a typical awl divided into thirds by height. d, diameter; A, area; V, volume. (B) Correlation matrix of the dimensional parameters of awl tip with the bone volume fraction (BV/TV) or bone surface density (BS/TV) of VOI1 and VOI2. (C) Correlation analysis of V1 with BV/TV or BS/TV of VOI1 and VOI2. (D) Correlation analysis of V2 with BV/TV or BS/TV of VOI1 and VOI2. (E) Correlation analysis of V3 with BV/TV or BS/TV of VOI1 and VOI2. (F) Correlation analysis of V1+V2+V3 with BV/TV or BS/TV of VOI1 and VOI2.


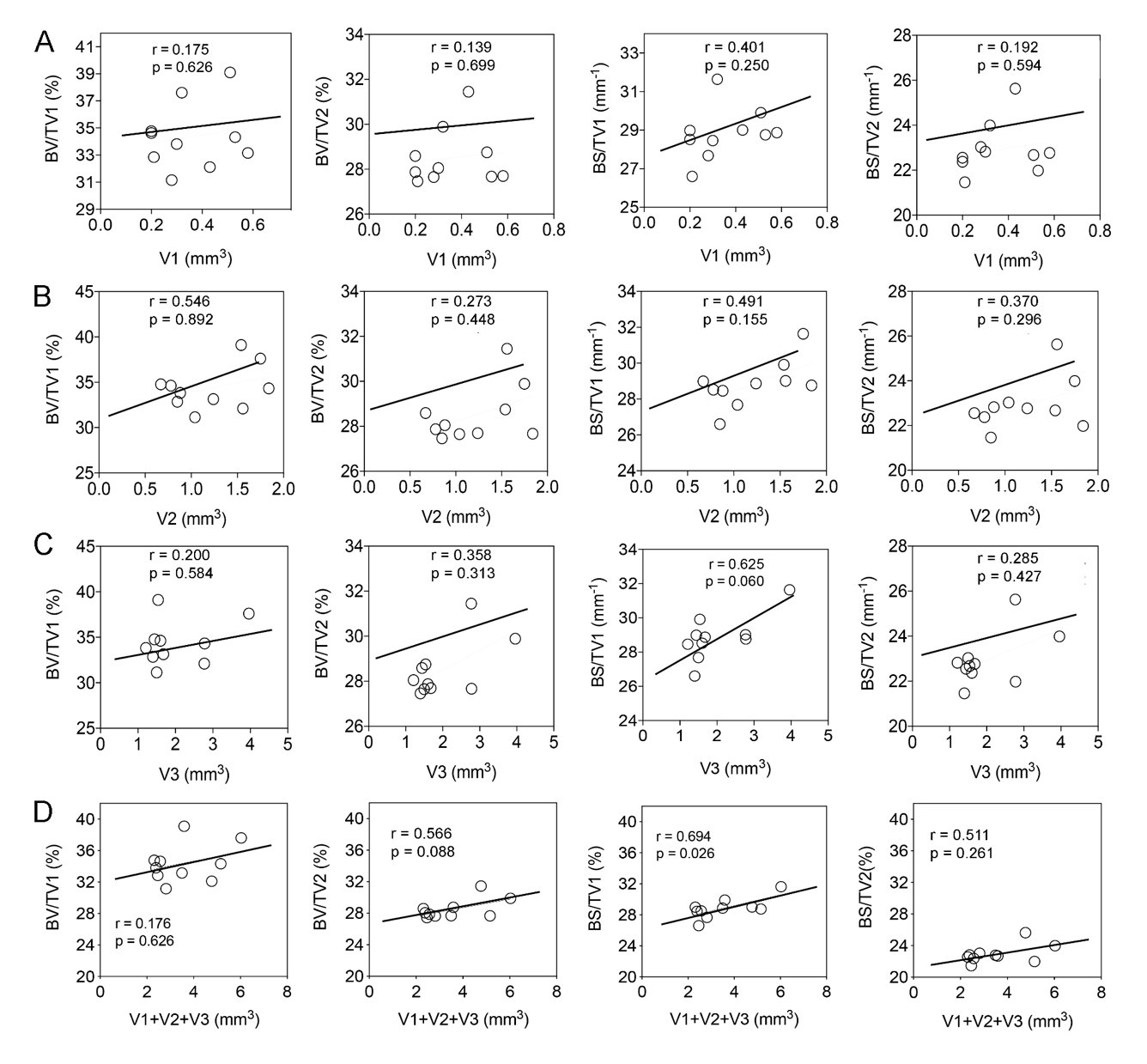


**TABLE S1**. Measurement of the parameters of the microfracture awls and K-wire.

| **Instrument name** | **Tip shape and angle** | **Base diameter (mm)** | | | **Base area (mm^2^)** | | | **Tip volume (mm^3^)** | | | |
| --- | --- | --- | --- | --- | --- | --- | --- | --- | --- | --- | --- |
|  |  | **D1** | **D2** | **D3** | **A1** | **A2** | **A3** | **V1** | **V2** | **V3** | **V1+V2+V3** |
| Atlantech awl | Cone-shaped; 30° | 1.07 | 1.50 | 1.75 | 0.90 ± 0.05 | 1.77 ± 0.08 | 2.39 ± 0.10 | 0.30 ± 0.02 | 0.88 ± 0.04 | 1.21 ± 0.05 | 2.39 ± 0.03 |
| Arthrex awl | Cone-shaped; 20° | 1.43 | 2.13 | 2.56 | 1.60 ± 0.09 | 3.56 ± 0.10 | 5.15 ± 9.19 | 0.53 ± 0.03 | 1.84 ± 0.04 | 2.78 ± 0.04 | 5.16 ± 0.02 |
| RZ Medizintechnik awl | Cone-shaped; 30° | 0.89 | 1.42 | 1.77 | 0.63 ± 0.02 | 1.59 ± 0.04 | 2.46 ± 0.06 | 0.21 ± 0.01 | 0.85 ± 0.02 | 1.40 ± 0.04 | 2.46 ± 0.01 |
| Rudolf awl | Cone-shaped; 30° | 0.87 | 1.36 | 1.81 | 0.59 ± 0.03 | 1.46 ± 0.07 | 2.57 ± 0.09 | 0.20 ± 0.01 | 0.78 ± 0.03 | 1.60 ± 0.05 | 2.57 ± 0.03 |
| Aesculap awl | Cone-shaped; 30° | 1.29 | 1.95 | 2.46 | 1.30 ± 0.05 | 3.00 ± 0.08 | 4.77 ± 0.11 | 0.43 ± 0.02 | 1.56 ± 0.04 | 2.77 ± 0.06 | 4.77 ± 0.05 |
| Smith & Nephew small awl | Triangular pyramid-shaped; 30° | 1.03 | 1.59 | 1.89 | 0.83 ± 0.06 | 1.98 ± 0.09 | 2.82 ± 0.11 | 0.28 ± 0.02 | 1.04 ± 0.04 | 1.50 ± 0.05 | 2.82 ± 0.04 |
| Smith & Nephew large awl | Cone-shaped; 30° | 1.49 | 1.87 | 2.11 | 1.75 ± 0.03 | 2.74 ± 0.02 | 3.50 ± 0.02 | 0.58 ± 0.01 | 1.24 ± 0.00 | 1.68 ± 0.00 | 3.50 ± 0.01 |
| Linvatec ConMed light awl | Cone-shaped; 30° | 0.87 | 1.29 | 1.71 | 0.59 ± 0.25 | 1.30 ± 0.40 | 2.31 ± 0.57 | 0.20 ± 0.08 | 0.67 ± 0.18 | 1.44 ± 0.30 | 2.31 ± 0.05 |
| Linvatec ConMed heavy awl | Cone-shaped; 30° | 1.11 | 1.99 | 2.77 | 0.97 ± 0.02 | 3.11 ± 0.08 | 6.03 ± 0.12 | 0.32 ± 0.01 | 1.75 ± 0.05 | 3.96 ± 0.07 | 6.03 ± 0.05 |
| CL Medical Kirschner wire | Triangular pyramid-shaped; 0° | 1.40 | 1.40 | 1.40 | 1.54 ± 0.00 | 1.54 ± 0.00 | 1.54 ± 0.00 | 0.51 ± 0.00 | 1.54 ± 0.00 | 1.54 ± 0.00 | 3.59 ± 0.00 |

Parameters were measured of parts at distance of 3 mm to the tip of the instruments. Base area and tip volume of all instruments, except for the Kirschner wire, are the average of 3 measurements are shown. Data shown in mean ± standard deviation. D, base diameter; A, base area; V, volume.

**TABLE S2.** Intergroup comparison of total volume of the instrument tips. **P* < 0.05.

| **Primary Instrument (PI)** | **Other Instruments (OI)** | **Mean Difference (PI-OI)** | **95% Confidence Interval** | | ***P* values** |
| --- | --- | --- | --- | --- | --- |
|  |  |  | **Lower Bound** | **Upper Bound** |  |
| Atlantech awl | Arthrex awl | -2.76 | -3.34 | -2.19 | <0.001* |
|  | RZ Medizintechnik awl | -0.07 | -0.65 | 0.51 | 1.000 |
|  | Rudolf awl | -0.18 | -0.76 | 0.40 | 0.980 |
|  | Aesculap awl | -2.38 | -2.95 | -1.80 | <0.001* |
|  | Smith & Nephew small awl | -0.42 | -1.00 | 0.15 | 0.278 |
|  | Smith & Nephew large awl | -1.11 | -1.69 | -0.53 | <0.001* |
|  | Linvatec ConMed light awl | 0.09 | -0.49 | 0.67 | 1.000 |
|  | Linvatec ConMed heavy awl | -3.64 | -4.22 | -3.06 | <0.001* |
|  | CL Medical Kirschner wire | -1.20 | -1.77 | -0.62 | <0.001* |
| Arthrex awl | Atlantech awl | 2.76 | 2.19 | 3.34 | <0.001* |
|  | RZ Medizintechnik awl | 2.69 | 2.12 | 3.27 | <0.001* |
|  | Rudolf awl | 2.58 | 2.01 | 3.16 | <0.001* |
|  | Aesculap awl | 0.39 | -0.19 | 0.96 | 0.388 |
|  | Smith & Nephew small awl | 2.34 | 1.76 | 2.92 | <0.001* |
|  | Smith & Nephew large awl | 1.65 | 1.08 | 2.23 | <0.001* |
|  | Linvatec ConMed light awl | 2.85 | 2.27 | 3.43 | <0.001* |
|  | Linvatec ConMed heavy awl | -0.88 | -1.45 | -0.30 | 0.001* |
|  | CL Medical Kirschner wire | 1.57 | 0.99 | 2.14 | <0.001* |
| RZ Medizintechnik awl | Atlantech awl | 0.07 | -0.51 | 0.65 | 1.000 |
|  | Arthrex awl | -2.69 | -3.27 | -2.12 | <0.001* |
|  | Rudolf awl | -0.11 | -0.69 | 0.47 | 0.999 |
|  | Aesculap awl | -2.31 | -2.88 | -1.73 | <0.001* |
|  | Smith & Nephew small awl | -0.36 | -0.93 | 0.22 | 0.498 |
|  | Smith & Nephew large awl | -1.04 | -1.62 | -0.46 | <0.001* |
|  | Linvatec ConMed light awl | 0.16 | -0.42 | 0.74 | 0.991 |
|  | Linvatec ConMed heavy awl | -3.57 | -4.15 | -2.99 | <0.001* |
|  | CL Medical Kirschner wire | -1.13 | -1.70 | -0.55 | <0.001* |
| Rudolf awl | Atlantech awl | 0.18 | -0.40 | 0.76 | 0.980 |
|  | Arthrex awl | -2.58 | -3.16 | -2.01 | <0.001* |
|  | RZ Medizintechnik awl | 0.11 | -0.47 | 0.69 | 0.999 |
|  | Aesculap awl | -2.20 | -2.77 | -1.62 | <0.001* |
|  | Smith & Nephew small awl | -2.25 | -0.82 | 0.33 | 0.874 |
|  | Smith & Nephew large awl | -0.93 | -1.51 | -0.35 | <0.001* |
|  | Linvatec ConMed light awl | 0.27 | -0.31 | 0.84 | 0.814 |
|  | Linvatec ConMed heavy awl | -3.46 | -4.04 | -2.88 | <0.001* |
|  | CL Medical Kirschner wire | -1.02 | -1.59 | -0.44 | <0.001* |
| Aesculap awl | Atlantech awl | 2.38 | 1.80 | 2.95 | <0.001* |
|  | Arthrex awl | -0.39 | -0.96 | 0.19 | 0.388 |
|  | RZ Medizintechnik awl | 2.31 | 1.73 | 2.88 | <0.001* |
|  | Rudolf awl | 2.20 | 1.62 | 2.77 | <0.001* |
|  | Smith & Nephew small awl | 1.95 | 1.37 | 2.53 | <0.001* |
|  | Smith & Nephew large awl | 1.27 | 0.69 | 1.84 | <0.001* |
|  | Linvatec ConMed light awl | 2.46 | 1.89 | 3.04 | <0.001* |
|  | Linvatec ConMed heavy awl | -1.26 | -1.84 | -0.69 | <0.001* |
|  | CL Medical Kirschner wire | 1.18 | 0.60 | 1.76 | <0.001* |
| Smith & Nephew small awl | Atlantech awl | 0.42 | -0.15 | 1.00 | 0.278 |
|  | Arthrex awl | -2.34 | -2.92 | -1.76 | <0.001* |
|  | RZ Medizintechnik awl | 0.36 | -0.22 | 0.93 | 0.498 |
|  | Rudolf awl | 0.25 | -0.33 | 0.82 | 0.874 |
|  | Aesculap awl | -1.95 | -2.53 | -1.37 | <0.001* |
|  | Smith & Nephew large awl | -0.69 | -1.26 | -0.11 | 0.012* |
|  | Linvatec ConMed light awl | 0.51 | -0.06 | 1.09 | 0.108 |
|  | Linvatec ConMed heavy awl | -3.21 | -3.79 | -2.64 | <0.001* |
|  | CL Medical Kirschner wire | -0.77 | -1.35 | -0.19 | 0.004* |
| Smith & Nephew large awl | Atlantech awl | 1.11 | 0.53 | 1.69 | <0.001* |
|  | Arthrex awl | -1.65 | -2.23 | -1.08 | <0.001* |
|  | RZ Medizintechnik awl | 1.04 | 0.46 | 1.62 | <0.001* |
|  | Rudolf awl | 0.93 | 0.35 | 1.51 | <0.001* |
|  | Aesculap awl | -1.27 | -1.84 | -0.69 | <0.001* |
|  | Smith & Nephew small awl | 0.69 | 0.11 | 1.26 | 0.012* |
|  | Linvatec ConMed light awl | 1.20 | 0.62 | 1.78 | <0.001* |
|  | Linvatec ConMed heavy awl | -2.53 | -3.11 | -1.95 | <0.001* |
|  | CL Medical Kirschner wire | -0.09 | -0.66 | 0.49 | 1.000 |
| Linvatec ConMed light awl | Atlantech awl | -0.09 | -0.67 | 0.49 | 1.000 |
|  | Arthrex awl | -2.85 | -3.43 | -2.27 | <0.001* |
|  | RZ Medizintechnik awl | -0.16 | -0.73 | 0.42 | 0.991 |
|  | Rudolf awl | -0.27 | -0.84 | 0.31 | 0.814 |
|  | Aesculap awl | -2.46 | -3.04 | -1.89 | <0.001* |
|  | Smith & Nephew small awl | -0.51 | -1.09 | 0.06 | 0.108 |
|  | Smith & Nephew large awl | -1.20 | -1.78 | -0.62 | <0.001* |
|  | Linvatec ConMed heavy awl | -3.73 | -4.30 | -3.15 | <0.001* |
|  | CL Medical Kirschner wire | -1.28 | -1.86 | -0.71 | <0.001* |
| Linvatec ConMed heavy awl | Atlantech awl | 3.64 | 3.06 | 4.22 | <0.001* |
|  | Arthrex awl | 0.88 | 0.30 | 1.45 | 0.001* |
|  | RZ Medizintechnik awl | 3.57 | 2.99 | 4.15 | <0.001* |
|  | Rudolf awl | 3.46 | 2.88 | 4.04 | <0.001* |
|  | Aesculap awl | 1.26 | 0.69 | 1.84 | <0.001* |
|  | Smith & Nephew small awl | 3.21 | 2.64 | 3.79 | <0.001* |
|  | Smith & Nephew large awl | 2.53 | 1.95 | 3.11 | <0.001* |
|  | Linvatec ConMed light awl | 3.73 | 3.15 | 4.30 | <0.001* |
|  | CL Medical Kirschner wire | 2.44 | 1.87 | 3.02 | <0.001* |
| CL Medical Kirschner wire | Atlantech awl | 1.20 | 0.62 | 1.77 | <0.001* |
|  | Arthrex awl | -1.57 | -2.14 | -0.99 | <0.001* |
|  | RZ Medizintechnik awl | 1.13 | 0.55 | 1.70 | <0.001* |
|  | Rudolf awl | 1.02 | 0.44 | 1.59 | <0.001* |
|  | Aesculap awl | -1.18 | -1.76 | -0.60 | <0.001* |
|  | Smith & Nephew small awl | 0.77 | 0.19 | 1.35 | 0.004* |
|  | Smith & Nephew large awl | 0.09 | -0.49 | 0.66 | 1.000 |
|  | Linvatec ConMed light awl | 1.28 | 0.71 | 1.86 | <0.001* |
|  | Linvatec ConMed heavy awl | -2.44 | -3.02 | -1.87 | <0.001* |

**TABLE S3**. Comparisons of bone volume fraction (BV/TV) and bone surface density (BS/TV) of the subchondral bone among VOI1, VOI2, and normal control.

| **Instrument name** | **BV/TV** | | | | | **BS/TV** | | | |
| --- | --- | --- | --- | --- | --- | --- | --- | --- | --- |
|  | **VOI1** | **VOI2** | **Normal control** | ***P* value** | **VOI1** | | **VOI2** | **Normal control** | ***P* value** |
| Atlantech awl | 33.82 ± 5.33 | 28.05 ± 3.78 | 24.39 ± 1.42 | # | 28.46 ± 6.18 | | 22.82 ± 5.26 | 22.64 ± 1.69 | n.s. |
| Arthrex awl | 34.32 ± 8.41 | 27.67 ± 7.22 | 24.88 ± 1.96 | # | 28.76 ± 9.14 | | 21.98 ± 7.25 | 22.86 ± 1.43 | n.s. |
| RZ Medizintechnik awl | 32.85 ± 6.96 | 27.46 ± 6.48 | 30.23 ± 1.29 | # | 26.60 ± 6.17 | | 21.46 ± 5.64 | 30.12 ± 1.52 | n.s. |
| Rudolf awl | 34.63 ± 5.72 | 27.87 ± 5.68 | 31.95 ± 1.64 | * # | 28.52 ± 4.73 | | 22.38 ± 4.36 | 30.24 ± 0.68 | * # |
| Aesculap awl | 32.11 ± 7.03 | 31.45 ± 5.95 | 23.68 ± 0.74 | # | 29.01 ± 5.68 | | 25.63 ± 4.88 | 23.32 ± 1.50 | # |
| Smith & Nephew small awl | 31.15 ± 5.84 | 27.65 ± 5.90 | 28.65 ± 1.97 | n.s. | 27.68 ± 6.42 | | 23.03 ± 5.33 | 17.39 ± 1.41 | n.s. |
| Smith & Nephew large awl | 33.15 ± 6.33 | 27.70 ± 6.40 | 22.84 ± 3.01 | # | 28.87 ± 5.59 | | 22.77 ± 5.41 | 22.45 ± 1.49 | * |
| Linvatec ConMed light awl | 34.77 ± 6.58 | 28.59 ± 6.71 | 25.31 ± 2.53 | # | 28.99 ± 5.71 | | 22.56 ± 5.74 | 23.43 ± 2.69 | * |
| Linvatec ConMed heavy awl | 37.61 ± 6.71 | 29.89 ± 6.40 | 26.06 ± 1.30 | * # | 31.64 ± 5.98 | | 23.99 ± 5.61 | 23.35 ± 1.55 | * # |
| CL Medical Kirschner wire | 39.10 ± 8.67 | 28.75 ± 5.94 | 19.88 ± 2.24 | * # | 29.91 ± 6.08 | | 22.68 ± 5.02 | 19.34 ± 2.09 | * # |

** P* < 0.05 for VOI1 *versus* VOI2; ^#^*P* < 0.05 for VOI1 *versus* normal control; ^§^*P* < 0.05 for VOI2 *versus* normal control. n.s., not significant.

**Reference**

1. Bakitian F, Seweryniak P, Papia E, Larsson C, Vult von Steyern P. Load-Bearing Capacity of Monolithic Zirconia Fixed Dental Prostheses Fabricated with Different Connector Designs and Embrasure Shaping Methods. *J Prosthodont.* 2019;28(1):64-70.

2. Hoemann CD, Gosselin Y, Chen H, et al. Characterization of initial microfracture defects in human condyles. *J Knee Surg.* 2013;26(5):347-355.

3. Gao L, Orth P, Goebel LK, Cucchiarini M, Madry H. A novel algorithm for a precise analysis of subchondral bone alterations. *Sci Rep.* 2016;6:32982.

4. Gao L, Orth P, Müller-Brandt K, Goebel LK, Cucchiarini M, Madry H. Early loss of subchondral bone following microfracture is counteracted by bone marrow aspirate in a translational model of osteochondral repair. *Sci Rep.* 2017;7:45189.

5. Orth P, Peifer C, Goebel L, Cucchiarini M, Madry H. Comprehensive analysis of translational osteochondral repair: Focus on the histological assessment. *Prog Histochem Cytochem.* 2015;50(3):19-36.
